# The boundary of posterior to level V region and the theoretical feasibility of irradiation dose reduction of level Va in nasopharyngeal carcinoma

**DOI:** 10.1038/s41598-024-52857-z

**Published:** 2024-01-28

**Authors:** Chaoyang Jiang, Xiaokai Li, Ling Zhang, Baolin Gong, Hui Gao, Zhihui Li, Tao Zhang, Juan Wang

**Affiliations:** 1 Department of Oncology, The General Hospital of Western Theater Command, Chengdu, 610083 Sichuan Province China; 2 Department of Nuclear Medicine, The General Hospital of Western Theater Command, Chengdu, 610083 Sichuan Province China

**Keywords:** Radiotherapy, Head and neck cancer

## Abstract

The lymph node involvement in the posterior to level V (PLV) region is mainly observed in nasopharyngeal carcinoma (NPC). Recently, we have reported the distribution of metastatic lymph nodes in the PLV region and there are correlations between the neck node levels (NNL) of NPC, but what is the boundary of the PLV region and how to delineate it remains unclear, and we further to elaborate whether the bilateral level Va should be covered as intermediate-risk nodal regions (CTVn2, about 60 Gy equivalent) for all T and N categories based on these correlations. A total of 1021 consecutive NPC patients with N1-3 stage from January 2012 to December 2020 were reviewed. The lymph node metastasis level of each patient was evaluated according to the updated guidelines proposed in 2013. According to the distribution pattern of lymph node metastasis and the anatomical structure in the PLV region, the boundaries of PLV region was delineated, and whether it is appropriate to cover the bilateral level Va as CTVn2 for all the NPC patients was further discussed. The correlations of level Va with other NNL were studied using logistic regression model. The cranial boundary of PLV region is the caudal border of cricoid cartilage, the caudal boundary is the plane serratus anterior muscle begins to appear, the anterior boundary is the anterior border of trapezius, and the posterior boundary is the convergence of levator scapulae and trapezius. Laterally, the PLV region is limited by the medial edge of trapezius and medially by the lateral surface of levator scapulae. The nodal spread in level Va is based on the lymph node metastasis of level IIb in NPC. The PLV region is a missing NNL of head and neck tumors, especially in NPC. The proposed boundaries of the PLV region can provide a preliminary proposal for the further revision of NNL in head and neck tumors. It is theoretically feasible to reduce the prophylactic irradiation dose of the bilateral level Va in patients with N0 stage or with isolated metastases in level VIIa.

## Introduction

**Figure 1:**
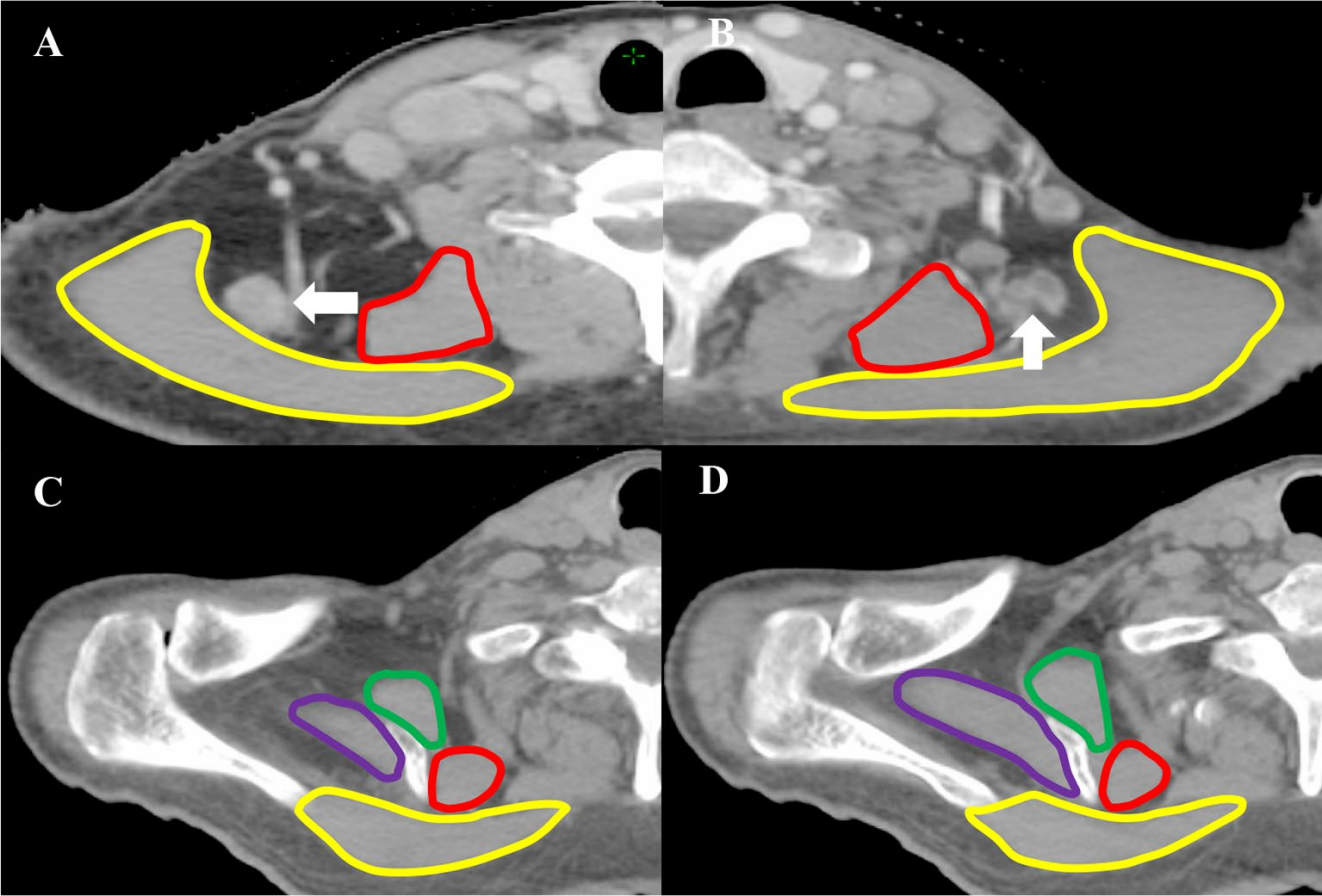
The yellow line represents trapezius, the red line represents levator scapulae, the green line presents the serratus anterior muscle, and the purple line presents the supraspinatus. (**A**, **B**) The PLV region in the CT scans, which is between levator scapulae and trapezius. The white arrows indicate the metastatic lymph nodes. (**C**, **D**) There is no space between levator scapulae and trapezius when the serratus anterior muscle appears on the CT image.

Nasopharyngeal carcinoma is a malignant tumor with a high rate of cervical lymph node metastasis^1–3^. Grégoire V et al. reported the recommendation for delineation of neck node target volumes based on the surgical experience in 2000^4^. A set of consensus guidelines for the delineation of NNL in node negative patients was published in 2003^5^, but not all the NNL were described in this consensus. A new consensus of the delineation of NNL for head and neck tumors was proposed in 2013 and accepted throughout the world (referred to as the 2013 guidelines)^6^. In the 2013 guidelines, 10 nodal groups were defined in accordance with the TNM atlas for cervical lymph nodes. However, there is no description of PLV region in the 2013 guidelines, and the PLV region is located in a deep gap between levator scapulae and trapezius. The metastatic lymph nodes can be observed in this region of some NPC patients (Fig. 1). Several studies have reported the metastasis rate of PLV region is 1.3–4.9%^7–9^. Unfortunately, the PLV region is often overlooked. We have reported the correlations between the PLV region and other NNL, the distribution of metastatic lymph nodes in the PLV region in our previous studies^9,10^, but what is the boundary of PLV region and how to delineate it is still unclear.

The international guideline recommended covering the bilateral level Va as CTVn2 for all the NPC patients^11^. Wang et al. had reported the lymph node metastasis rate of level Va in NPC is 21.5%^7^. Do we really need to cover the bilateral level Va of all the NPC patients within CTVn2 just because there are 21.5% of the patients have lymph node involvement in level Va? and there is no report.

This study aims to describe the boundaries of PLV region and to show how the PLV region is delineated. We hope to provide a preliminary proposal of boundary setting for the further revision of NNL in head and neck tumors. In addition, we further discuss whether it is appropriate to cover the bilateral level Va within CTVn2 for all the NPC patients.

## Methods

### Patient population

The study was retrospective in character and all methods were carried out in accordance with the ethical principles derived from the Declaration of Helsinki and its subsequent amendments (permission was obtained from the medical ethics committee of The General Hospital of Western Theater Command, the informed consent was exempted due to the retrospective nature of the study). There are 13 independent variables in our manuscript, and the lymph node metastasis rate of level Va is 21.5%^7^. The sample size was calculated by using the PASS software package (version 15.0, Power: 0.9, Alpha: 0.05, P0: 0.027, P1: 0.25) and the result is about 138 cases. We enrolled 1021 NPC patients whose records were retrospectively reviewed. The inclusion criterion was the patients had been pathologically confirmed as having NPC with any T and N1-3 stage according to the 8th edition of the AJCC/UICC staging system for NPC^12^, the tumor stages were also classified according to this staging system, and the exclusion criterion was the patients with N0 stage. All the patients were performed MRI scan before treatment.

### The diagnostic criteria of lymph node involvement

All the MRI and contrast-enhanced CT images of the patients were reviewed. The metastatic NNL for each patient were assessed according to the 2013 guidelines^6^. The diagnosis of lymph node metastasis was evaluated according to the following criteria^13^: (1) minimal axial diameter ≥ 5 mm in the retropharyngeal region, and ≥ 10 mm for all other cervical nodes. (2) Any size of the lymph node with necrosis or ring enhancement. (3) Any size of the lymph node with extracapsular invasion or fusion. (4) More than three contiguous lymph nodes with the shortest diameter of 8–10 mm. We also referred to the PET/CT images if the patients underwent this examination.

### Delineation of the boundaries in PLV region

The contrast-enhanced CT simulation images of an NPC patient with N0 stage were obtained before treatment. According to the distribution pattern of nodal spread and the anatomical structure in the PLV region, we delineated the cranial boundary, the caudal boundary, the anterior boundary, the posterior boundary, the lateral and medial boundary of PLV region on these CT images, respectively.

### Ethical approval and consent to participate

As the images in our manuscript were obtained from patients, the presented study was reviewed and approved by the medical ethics committee of The General Hospital of Western Theater Command.

The medical ethics committee of the General Hospital of Western Theater Command approved the study (2023EC2-KY003). The informed consent was exempted due to the retrospective nature of the study. All methods were carried out in accordance with ethical principles derived from the Declaration of Helsinki and its subsequent amendments.

### Statistical analysis

The logistic regression model was used to evaluate the correlations between level Va nodes and other NNL, and the exact conditional test was used for the quasi-complete separation of data points. The Hosmer–Lemeshow test was used to clarify the goodness of fit in logistic regression (*p* value > 0.05 showed good estimation of logistic model). Levels with less than a 3% metastatic rate were excluded in the logistic regression to avoid the bias. The SPSS 20.0 software and SAS software (SAS Institute Inc., Cary, NC, USA) was used to analyzed the data, statistical significance was defined as *p* < 0.05.

## Results

### Patient characteristics

There were 738 males and 283 females among 1021 NPC patients. The number of patients with T1, T2, T3, and T4 stage is 294 (28.8%), 198 (19.4%), 282 (27.6%), and 247 (24.2%), respectively. The number of patients with N1, N2, and N3 stage is 381 (37.3%), 452 (44.3%), and 188 (18.4%), respectively. The number of patients with stage II, III, IVa, and IVb is 192 (18.8%), 421 (41.2%), 389 (38.1%), and 19 (1.9%), respectively. All the patients’ baseline characteristics are listed in Table 1.

**Table 1:** Baseline characteristics of the NPC patients.

Characteristic	Number of patients (%)
Gender	
Male	738 (72.3%)
Female	283 (27.7%)
Age (year)	
Median	49
Range	12–85
T stage	
T1	294 (28.8%)
T2	198 (19.4%)
T3	282 (27.6%)
T4	247 (24.2%)
N stage	
N1	381 (37.3%)
N2	452 (44.3%)
N3	188 (18.4%)
TNM stage	
II	192 (18.8%)
III	421 (41.2%)
IVa	389 (38.1%)
IVb	19 (1.9%)

### The patterns of lymph node involvement

The most common levels of lymph node metastasis are VIIa (86.2%), IIb (84.6%), and the metastasis rate of other NNL are as follows: IIa (62.6%), III (48.9%), Va (27.8%), IVa (16.8%), Vb (7.6%), Ib (5.9%), and PLV region (5.2%). The rate of lymph node involvement in levels IVb, VIIb, and VIII is less than 5.0%, and there is no nodal spread in levels Ia, VI, IX, and X. The patterns of lymph node involvement are shown in Supplementary Table 1.

### The boundaries of PLV region

**Figure 2:**
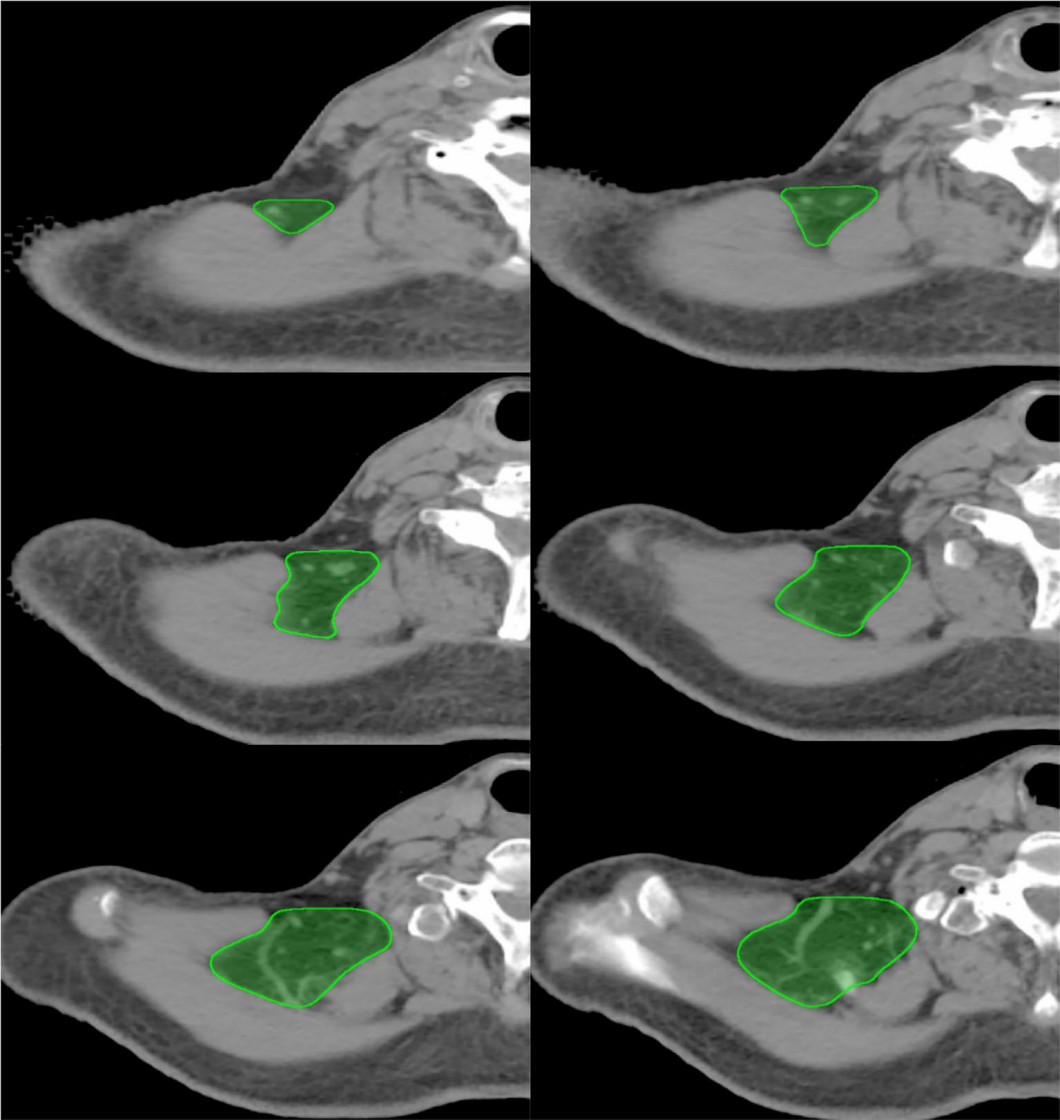
A set of CT scans to show how the PLV region is delineated, the green line represents the PLV region.

**Figure 3:**
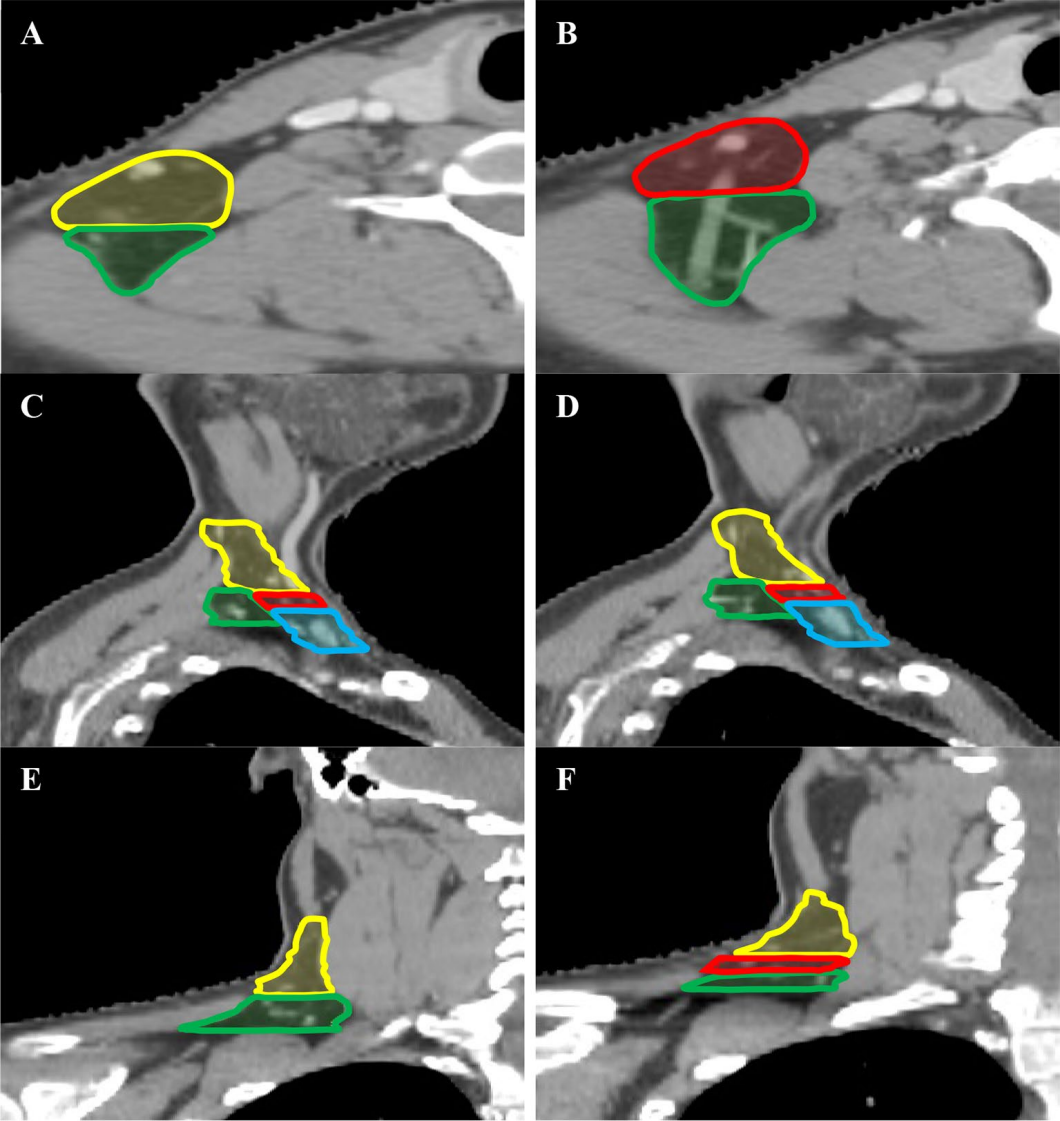
The PLV region and level V(a, b, c) was delineated in the axial, sagittal and coronal planes. The yellow line represents level Va, the red line represents level Vb, the blue line represents level Vc, the green line represents PLV region. (**A**, **B**) The axial planes. (**C**, **D**) The sagittal planes. (**E**, **F**) The coronal planes.

The cranial boundary of PLV region is the caudal border of cricoid cartilage, the caudal boundary is the plane serratus anterior muscle begins to appear, the anterior boundary is the anterior border of trapezius, and the posterior boundary is the convergence of levator scapulae and trapezius. Laterally, the PLV region is limited by the medial edge of trapezius and medially by the lateral surface of levator scapulae. The boundaries of PLV region are summarized in Table 2. A set of CT scans are provided to show how the PLV region is delineated in Figs. 2 and 3.

**Table 2:** The boundaries of PLV region.

PLV region	Boundaries					
	Cranial	Caudal	Anterior	Posterior	Lateral	Medial
	The caudal border of cricoid cartilage	The plane serratus anterior muscle begins to appear	The anterior border of trapezius	The convergence of levator scapulae and trapezius	The medial edge of trapezius	The lateral surface of levator scapulae

### The number of patients with or without nodal spread in level Va simultaneously accompanied with nodal involvement in other NNL

The number of patients with or without nodal spread in level Va is 284 (27.8%) and 737 (72.2%), respectively. Among 61 patients with nodal involvement in level Ib, there are 37 (3.6%) and 24 (2.4%) patients accompanied with or without nodal spread in level Va, respectively. As calculated in this way, there are 236 (23.1%) and 403 (39.5%) patients in level IIa accompanied with or without nodal spread in level Va, there are 284 (27.8%) and 580 (56.8%) patients in level IIb accompanied with or without nodal spread in level Va. All the data are summarized in Table 3.

**Table 3:** The number of patients with or without nodal spread in level Va simultaneously accompanied with nodal involvement in other NNL.

	Level Va metastasis at diagnosis		
	Variable	(−), n = 737	(+), n = 284
Level Ib involvement	No	713	247
	Yes	24	37
Level IIa involvement	No	334	48
	Yes	403	236
Level IIb involvement	No	157	0
	Yes	580	284
Level III involvement	No	488	34
	Yes	249	250
Level IVa involvement	No	698	151
	Yes	39	133
Level IVb involvement	No	732	252
	Yes	5	32
Level VIIa involvement	No	113	28
	Yes	624	256
Level VIIb involvement	No	731	283
	Yes	6	1
Level VIII involvement	No	735	272
	Yes	2	12

### The correlations of level Va nodes with other NNL

There are 284 (27.8%) patients had nodal involvement in level Va. The *p* value of Hosmer–Lemeshow test was 0.807. The logistic regression analysis indicated level Va was correlated with level IIb (*p* < 0.0001, OR 1.519, 95% CI 0.765–+ infinity), III (*p* < 0.0001, OR 4.376, 95% CI 2.784–6.877), IVa (*p* < 0.0001, OR 3.437, 95% CI 2.089–5.655), Vb (*p* < 0.0001, OR 2.099, 95% CI 1.341–+ infinity), and PLV region (*p* < 0.0001, OR 2.071, 95% CI 1.269–+ infinity). The results were summarized in Supplementary Table 2.

## Discussion

Nasopharyngeal carcinoma is one of head and neck tumors with a high rate of lymph node involvement^14–16^. Approximately 85% of the NPC patients were confirmed as having lymph node metastasis^7,17^. The PLV region is located in a deep gap between levator scapulae and trapezius, the metastatic lymph nodes can be observed in this region of some NPC patients, and the metastasis rate is 1.3–4.9%^7–9^, which was close to the metastasis rate of levels Ib and IVb in our study. However, there is no description of PLV region in the 2013 guidelines, which is a missing NNL for the head and neck tumors, especially in NPC. We think the accurate definition of PLV region can make the delineation of clinical target volumes (CTV) more accurately. The PLV region receives efferent lymphatics primarily from level V, the posterior neck and shoulder, and the lymph node involvement of PLV region is mainly associated with NPC. The PLV region extends from the anterior border of trapezius anteriorly to the convergence of levator scapulae and trapezius posteriorly, and from the caudal border of cricoid cartilage cranially to the plane serratus anterior muscle begins to appear caudally. We set the caudal border of cricoid cartilage as the cranial boundary of PLV region is because the space between levator scapulae and trapezius begins to appear at this plane. There is no space between levator scapulae and trapezius when the serratus anterior muscle begins to appear on the CT image, which we think is the caudal boundary of PLV region (Fig. 1), and we observed none of the involved lymph nodes in PLV region extended beyond this plane. The posterior boundary of levels Va and Vb is the anterior border of trapezius in the 2013 guidelines, which is the anterior boundary of PLV region in our study. There is no space behind the convergence of levator scapulae and trapezius, which is the posterior boundary of PLV region. The lateral and medial boundary of PLV region is the medial edge of trapezius and the lateral surface of levator scapulae, respectively. We observed all the involved lymph nodes in PLV region were distributed within the boundary range that we set. We must emphasize that the preliminary boundary setting of PLV region is based on the distribution pattern of nodal spread and the anatomical structure in this area from the 1021 NPC patients.

The 2013 guidelines subdivided level V into level Va, Vb, and Vc. We have reported all the correlations for the NNL of NPC in our previous study^10^. Unfortunately, we didn’t further elaborate whether level Va can be considered as CTVn2 for all NPC patients base on these correlations. Our study showed that the nodal involvement in level Va was related to levels IIb, III, IVa, Vb, and PLV region, but have no correlation with level VIIa. We observed all the patients with nodal involvement in level Vb or level Vc simultaneously had lymph node metastasis in level Va in our study, and all the 284 patients with nodal spread in level Va had lymph node metastasis in level IIb, there was no case with nodal involvement in level Va among the 157 patients who had no lymph node metastasis in level IIb (Table 3). A total of 144 patients had lymph node metastasis in level VIIa but without nodal involvement in level IIb in our study, and none of the 144 patients presented with nodal spread in level Va. There was no case who presented with lymph node involvement only in levels VIIa and Va in our study among the 1021 patients, and we didn’t observe a patient with isolated metastases in level V. Another study reported there was only 1 (0.1%, 1/924) NPC patient had nodal involvement in level V but without nodal spread in retropharyngeal region or level II^17^. Chone CT et al. had reported the histopathologic metastases in level II and III for all neck dissections are risk factors for metastases in the apex of the posterior triangle (the upper part of level V) in 51 patients with squamous cell carcinoma of head and neck tumors, and there was no case who only had lymph node metastasis in the upper part of level V^18^. Hamoir M et al. had reported the apex of level V (level Va) includes the drainage from occipital region and contains only superficial suprafascial occipital lymph nodes, the subfascial and submuscular lymph nodes located close to the occipital attachment of the sternocleidomastoid muscle^19^. From the anatomical structure in this region, the accessory nerve descends along the anterolateral side of the internal jugular vein after it exits the skull from the jugular foramen, and then passes through the anterior edge of the upper part of the sternocleidomastoid muscle to the deep surface of the trapezius muscle backward and downward. There are lymph nodes arranged along the accessory nerve, which means the lymph nodes can metastasize from level II to level V and PLV region along the accessory nerve, and this can explain why the nodal spread in level Va is mainly associated with level IIb and the lymph node metastasis in the PLV region is mainly associated with level Va. Therefore, the nodal spread in level Va is based on the lymph node involvement of level IIb in NPC.

**Figure 4:**
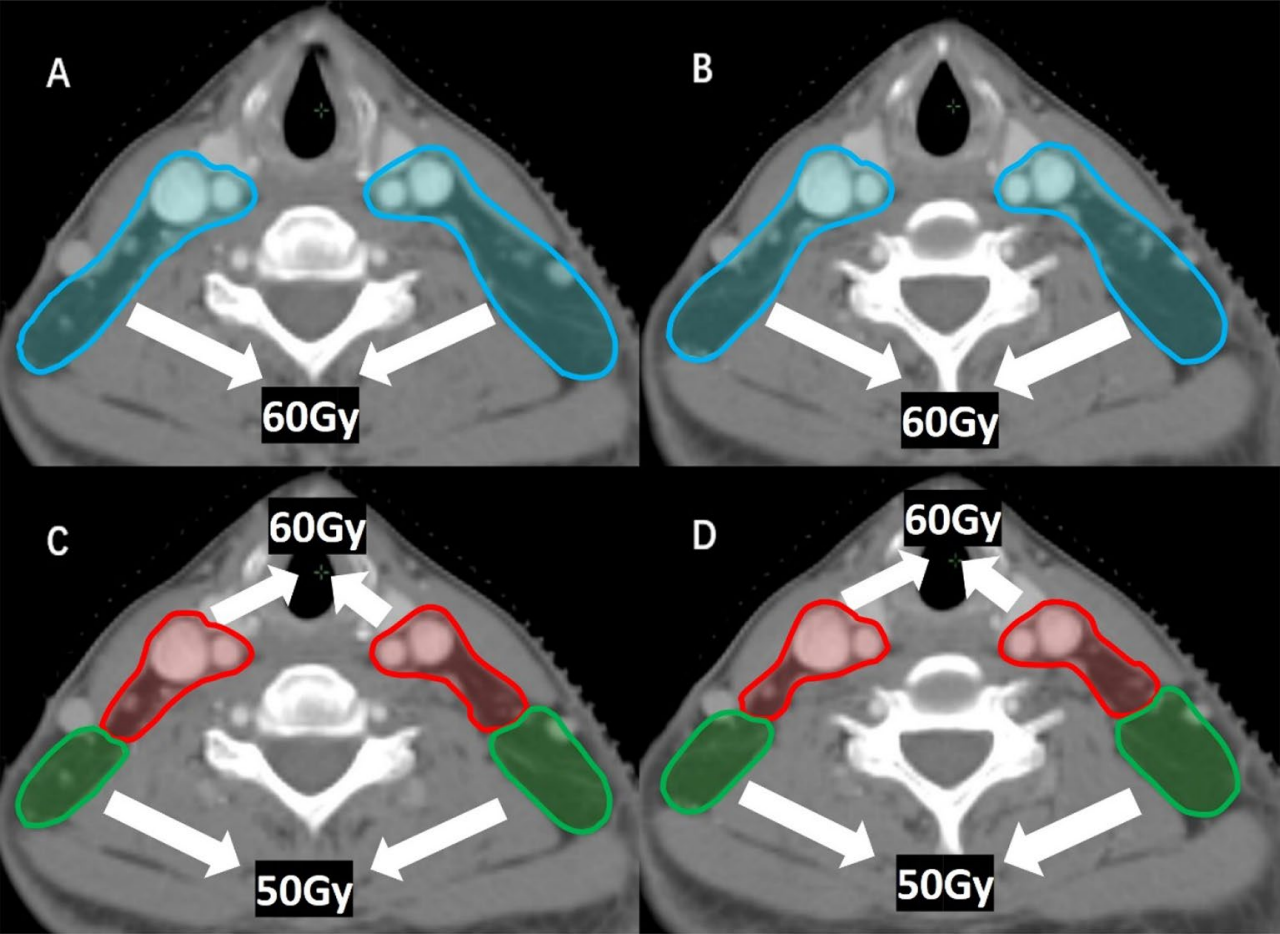
The CTV delineation of NPC patient with nodal involvement in patients with N0 stage or with isolated metastases in level VIIa. (**A**, **B**) The CTV delineation recommended by the international guideline (the blue line represents CTVn2). (**C**, **D**) The CTV delineation recommended by this study (the red line represents CTVn2 and the green line represents CTVn3).

Withers HR et al.had reported that the dose of 50 Gy can effectively control the potential lymph node involvement in the subclinical regions^20^, The rate of skip nodal involvement in NPC is quite low^17,21–23^, which means the lymph node metastasis of the uninvolved level is based on the involved levels and there are correlations between the NNL, so we just need to cover the NNL related to the involved levels rather than the whole neck as CTVn2, but the international guideline recommended covering the bilateral level Va as CTVn2 for all the NPC patients^11^. According to our analysis, there are about 15% of the NPC patients receive over prophylactic irradiation dose in the bilateral level Va because the rate of nodal spread in level IIb is about 85%, so it is inappropriate to perform high-dose prophylactic irradiation (about 60 Gy equivalent) on the bilateral level Va for all the NPC patients. Theoretically, the bilateral level Va can be considered as low-risk nodal regions (CTVn3, about 50 Gy equivalent) in patients with N0 stage or with isolated metastases in level VIIa (Fig. 4). We believe that it is beneficial for patients to reduce even 1 Gy of the prophylactic irradiation dose if the equivalent survival outcomes and regional control can be obtained, and high-dose prophylactic irradiation means the higher incidence of radioactive dermatitis and other dose-induced side effects. Furthermore, we consider the delineation of CTV based on the correlations of NNL rather than to cover the whole neck can reduce the prophylactic irradiation levels or dose of CTVn2.

There are some limitations in this study. First, the lymph nodes in PLV region have no histopathology, performing histopathological examination and lymph node dissection in this deep region is difficult and not recommended for NPC patients. Second, lymph node metastasis in PLV region was mainly observed in NPC patients, and we don’t perform a further study to confirm whether the patients also present with lymph node involvement in the PLV region in other head and neck tumors. Third, there is no follow-up data to support the conclusion because this is a retrospective study, but it is a strong theoretical support for carrying out prospective research, and we are conducting a prospective study of the CTV delineation based on this conclusion, we will report the results in the future.

## Conclusions

We report in detail the boundaries of PLV region and how to delineate it in NPC patients, which can provide a preliminary proposal for the further revision of NNL in head and neck tumors. We recommend using the PLV region as a new NNL. Theoretically, the bilateral level Va can be delineated as CTVn3 in patients with N0 stage or with isolated metastases in level VIIa.

### Supplementary Information

Supplementary Table 1.

Supplementary Table 2.

## Data Availability

The datasets used during the current study available from the corresponding author on reasonable request.

## Abbreviations

PLV: Posterior to level V region
NPC: Nasopharyngeal carcinoma
NNL: Neck node levels
CTV: Clinical target volumes
CTVn2: Intermediate-risk nodal regions
CTVn3: Low-risk nodal regions
