# Supplementary material for: The boundary of posterior to level V region and the theoretical feasibility of irradiation dose reduction of level Va in nasopharyngeal carcinoma
Source: Sci Rep. 2024 Jan 28;14:2308. doi: 10.1038/s41598-024-52857-z (PMC10821861; doi:10.1038/s41598-024-52857-z)
Supplement: Supplementary file 1 — Supplementary Table 1. [file 41598_2024_52857_MOESM1_ESM.docx]

**Supplementary table 1.** Patterns of cervical lymph node metastasis of NPC

| Neck node levels | Number of patients (%) |
| --- | --- |
| Ⅰa | 0(0.0) |
| Ⅰb | 61(5.9) |
| IIa | 639(62.6) |
| IIb | 864(84.6) |
| III | 499(48.9) |
| IVa | 172(16.8) |
| IVb | 37(3.6) |
| Va | 284(27.8) |
| Vb | 78 (7.6) |
| Vc | 25(2.4) |
| PLV | 53(5.2) |
| VI | 0(0.0) |
| VIIa | 880(86.2) |
| VIIb | 7(0.7) |
| VIII | 14(1.4) |
| IX | 0(0.0) |
| X | 0(0.0) |
