# Supplementary material for: The boundary of posterior to level V region and the theoretical feasibility of irradiation dose reduction of level Va in nasopharyngeal carcinoma
Source: Sci Rep. 2024 Jan 28;14:2308. doi: 10.1038/s41598-024-52857-z (PMC10821861; doi:10.1038/s41598-024-52857-z)
Supplement: Supplementary file 2 — Supplementary Table 2. [file 41598_2024_52857_MOESM2_ESM.docx]

**Supplementary table 2.** The results of logistic regression analysis of level Va and other NNL.

| Factor | *P value* | *OR* | *95% CI for OR* | |
| --- | --- | --- | --- | --- |
| Level Ib | 0.478 | 1.295 | 0.634 | 2.644 |
| Level IIa | 0.144 | 1.381 | 0.896 | 2.130 |
| Level IIb | **0.0001*#** | 1.519 | 0.765 | +infinity |
| Level III | **0.0001*** | 4.376 | 2.784 | 6.877 |
| Level IVa | **0.0001*** | 3.437 | 2.089 | 5.655 |
| Level IVb | 0.843 | 0.883 | 0.258 | 3.022 |
| Level Vb | **0.0001*#** | 2.099 | 1.341 | +infinity |
| Level VIIa | 0.551 | 1.187 | 0.676 | 2.083 |
| PLV region | **0.0001*#** | 2.071 | 1.269 | +infinity |

***** *p* < 0.0001, **#** The exact conditional test was used.
